# Does patient engagement affect IBD patients’ health-related quality of life? Findings from a cross-sectional study among people with inflammatory bowel diseases

**DOI:** 10.1186/s12955-021-01724-w

**Published:** 2021-03-07

**Authors:** Serena Barello, Elena Guida, Salvatore Leone, Enrica Previtali, Guendalina Graffigna

**Affiliations:** 1 EngageMinds Hub, Consumer, Food and Health Engagement Research Center, Faculty of Psychology, Università Cattolica del Sacro Cuore, Milan, Italy; 2 EngageMinds Hub, Consumer, Food & Health Engagement Research Center, Università Cattolica del Sacro Cuore, Milan, Italy; 3 Amici Onlus, Milan, Italy; 4 EngageMinds Hub, Consumer, Food & Health Engagement Research Center, Faculty of Agriculture, Food and Environmental Sciences, Università Cattolica del Sacro Cuore, Piacenza, Italy

**Keywords:** Patient engagement, Health-related quality of life, Inflammatory bowel diseases, Healthcare, PHE-s®

## Abstract

**Background:**

Patients diagnosed with inflammatory bowel disease (IBD) are required to deal with the unpredictability of this clinical condition, which is associated with poorer health-related quality of life (HRQoL) compared to other clinical conditions. Patient engagement is currently demonstrated to relate with chronic patients’ HRQoL, but few studies have been conducted among this population.

**Methods:**

A cross-sectional study was conducted among 1176 IBD patients. Data were collected on participants’ HRQoL (SIBD-Q) and patient engagement (PHE-s®). Regression analysis was used to examine the effects of patient engagement on HRQoL.

**Results:**

About the half of the sample (47%) reported a low patient engagement level. 30% of the sample reported a low level of HRQoL. Psycho-emotional functioning resulted to be the aspect of HRQoL most impacted in the 37% of the sample. The regression model showed that PHE-s® is significantly related to the SIBD-Q total score (B = .585; *p* < .001; R squared = .343) and to the subscales’ scores—systemic symptoms (B = .572; *p* < .001; R squared = .327), bowel symptoms (B = .482; *p* < .001; R squared = .232), social (B = .485; *p* < .001; R squared = .234) and psycho-emotional (B = .607; *p* < .001; R squared = .369) functioning.

**Conclusions:**

Patients who are engaged in their IBD care pathway are more likely to report higher level of HRQoL, thus offering clues to potential therapeutic approaches to ameliorating IBD patients’ wellbeing. As this is a modifiable factor, screening for patient  health engagement levels, coupled with appropriate interventions, could improve care, and ultimately improve HRQoL outcomes among IBD patients.

## Background

At the turn of the twenty-first century, Inflammatory Bowel Disease (IBD) has become a global disease with increased incidence in newly industrialised countries. The global epidemiology of IBD has changed substantially over time, with a recent review demonstrating an increasing incidence of inflammatory bowel disease in developing nations and a stabilising incidence in developed nations [1][1][1]. The incidence rate of Crohn’s disease (CD) in Italy is lower than those reported for other Mediterranean countries; in contrast, the Ulcerative Colitis (UC) incidence rate is within the range of those reported in European studies[4].

CD and UC are the two main conditions that collapse into IBD, that is an idiopathic disease of the gastrointestinal system that is characterized by chronic bowel inflammation [5]. They can affect any age group, have a significant impact on working age populations and lead to a considerable societal and economic burden. The goal of treatment for IBD is to diminish or eliminate disease activity and optimize health-related quality of life (HRQoL). Thus, healthcare services need to address any factors that might contribute to disease flares and diminished HRQoL [6, 7].

Patients diagnosed with IBD are required to deal with the uncertainty of the symptoms and the unpredictability of this clinical condition along their illness journey [6, 8]. Particularly, these factors can greatly inhibit their personal, social, employment, and recreational functioning, thus deeply impacting their HRQoL. For these reasons, beside medical and physical consequences, IBD can also have a significant negative impact on patients’ HRQoL. Factors that appear to affect the HRQoL of IBD patients include the disease course (extent, severity, and pattern of symptoms’ relapse)[9], prescribed therapy (efficacy, side effects, and burden of administration) [10], and non-disease-related factors[11–13]. Among non-disease related factors, an increasing body of literature is currently demonstrating the role psychosocial factors in affecting IBD patients’ HRQoL [12–16]. For instance, perceived stress has been found to be correlated to disease exacerbation, thus indirectly affecting patients’ HRQoL [17]. On the contrary, effective coping strategies help patients to more effectively adjust to adverse stressors, thus improving both clinical outcomes and satisfaction with life [11, 18]. On the other side, maladaptive coping behaviors such as self-blame, avoidance strategies are negatively correlated with patients’ HRQoL [11, 19, 20]. Other authors showed how self-image and the individuals’ ability to engage in intimate relationships – which are often compromised in patients with IBD—are important factors in their HRQoL [15]. Finally, some authors have also demonstrated the role of some personality traits in affecting the illness course [14, 21, 22]. In some studies, stable personality traits such as neuroticism and alexithymia were found to be related to IBD patients’ HRQoL, whereas social desirability was not [23, 24]. Another psychosocial aspect that research pointed out as worth to be investigated is that IBD patients’ adaptive attitudes towards the disease and its treatment make them more resilient and able to manage disease-related stressors [13, 25–28]. Particularly, among the adaptive attitudes that patients might enact, patient health engagement—described as the psychological readiness of individuals to become active player in their own healthcare management [29]—is more and more recognized to be a predictive factor of patient’s healthy behaviors and better clinical and psychological outcomes among chronic conditions such as IBD [30–37].

Goldring and colleagues, for instance, found that patients who were engaged in an active shared decision-making regarding their disease management with their doctor were more likely to report greater HRQoL[38]. Accordingly, Munson and colleagues provided some evidence that there are significant linkages between patients’ attitudes towards their care and their HRQoL [37]. Moreover, literature on chronic patients suggests that patients who report higher engagement in the care process are more likely to report better HRQoL scores [39–41].

However, to the best of our knowledge, no study has focused on the role of IBD patient health engagement in disease management in determining their HRQoL. According to these premises, with this study we aimed at detecting the role of the patients’ psychological readiness to be active player in their IBD management (i.e. patient health engagement) in affecting patient’s HRQoL.

## Methods

### Study design and population

A cross sectional online survey study has been conducted on a purposive, not representative sample of Italian patients diagnosed with IBD. The study was based on a semi-structured self-administered online questionnaire consisting of validated psychometrics scales and had hoc items. More details on the questionnaire are provided in the following section.

Recruited patients belong to an IBD national-based Italian patient organization named *“A.M.I.C.I. onlus”* (Italian National Federation of Inflammatory Bowel Disease). They were invited—using mailing list of the patient organization *A.M.I.C.I. onlus*—to take part to this study between November 2016 and December 2017 and to complete and online survey distributed through the online platform Qualtrics®. No incentives were given to participants. Inclusion criteria were: age 18 years and older, diagnosed with IBD, and being able to read and understand the questionnaire.

### Measures

The study questionnaire consisted of psychometric tools to address the research objectives. Moreover, we collected socio-demographics and clinical variables (age, gender, education, presence of children, employment, diagnosis, years from diagnosis, number of relapses). More details on the psychometric instruments implied in the study are provided in the following paragraphs.

### Patient health engagement: PHE-s Scale®

The Italian version of the PHE-s scale® [42] was used to evaluate patients’ level of individual’s engagement in their health care management. The scale is based on the PHE model [43] which is a developmental psychological theory describing the patient’s experience of becoming active players in their healthcare pathway. According to this model, individuals may be differently engaged in care management according to their emotional, cognitive, and behavioral mindset [42]. Across the patient health engagement journey, while patients gain knowledge and information about their disease, they generally become more emotionally adjected to their health condition and more confident in their ability to manage their disease.

The PHE-s scale® is able to grasp the complex psychological experience of the patient health engagement and has ordinal structure in order to be consistent with the PHE model's conceptualization, which, through an algorithm which provides the final score, envisages four different positions along the engagement continuum: Blackout, Alert, Adhesion and Eudaimonic Project [29]. Specifically, patients have been considered with a “low PHE level” when they are positioned in Blackout or Alert, whereas they were defined as “high PHE level” when their scoring was related to the Adhesion and Eudaimonic positions (see Table 2). The scale is measured on a 7-point scale in order to facilitate patients' responses and to avoid social desirability bias[42].

### Health-related quality of life: SIBD-Q

Patients’ HRQoL has been measured through the Short Inflammatory Bowel Disease Questionnaire (SIBD-Q) [44, 45]. SIBD-Q is a validated and reliable tool which measures HRQoL in adult patients with IBD, UC, or CD. It contains 32 questions, divided into four HRQoL domains: bowel symptomatology (10 questions), systemic symptoms (5 questions), psycho-emotional function (12 questions), and social functioning (5 questions). For each question there are graded responses on a 7-point Likert scale, ranging from 1 (representing the "worst" aspect) to 7 (representing the "best" aspect). The four HRQoL domains are calculated as the mean of the questions referring to each specific health area and. The total SIBD-Q score ranges from 32 to 224, with higher scores reflecting better wellbeing. Finally, patients have been categorized as with a “low level of HRQoL” or with a “high level of HRQoL” for each domain, depending on the mean rate they reported: if it was above the median point of the Likert scale, they were considered as “high”, while if it was below, they were in a “low” position.

### Statistical analysis

Statistical analysis has been run with the software IBM SPSS 23. Socio-demographic characteristic were analysed using descriptive and frequencies analysis. Preliminary, we tested the consistency of PHE-s® both as continuous and categorical variable and correlations were used to test any association between socio-demographic data and other variables. Secondarily, to understand whether PHE-s® has a statistically significant effect on SIBD-Q total score and subscales when confounding variables—i.e. patients’ age, year of diagnosis and number of relapses, education, employment and presence of children—are controlled, a MANCOVA has been performed. Finally, we explored the relation between PHE-s® and SIBD-Q trough a simple linear regression model to understand the predictive power of patients’ health engagement on IBD patients’ HRQoL.

### Ethical considerations

The study protocol was approved by the Ethics Commission of the Department of Psychology of the Catholic University of Milan (Italy). All patients involved in the study gave informed consent to participate.

## Results

The sample involved in this study consisted of 1176 IBD patients. All the subjects that was present in the mailing list of the patient organization were contacted to fill out the online questionnaire. No data about who did not fill out the questionnaire were available. Socio-demographic data of people that completed the survey are showed in Table 1 and highlight an almost equal distribution between male and female participants, between those with and without children and a mean age of 45 years old. 51% has a high school degree and 86% is currently employed. The average number of disease relapses during the previous year is of 2, for a minimum of 0 and a maximum of 5 (see Table 1). Participants were also asked to report the number of years from the IBD diagnosis which ranged from 1 to 47 year(s).

**Table 1:** Socio-Demographic and clinical characteristics of the sample (N = 1176)

	Total		Chron's disease		Ulcerative colitis		Unclassified colitis	
	N	%	N	%	N	%	N	%
*Gender*								
Female	365	48.3%	189	25,0%	171	22,6%	2	0,3%
Male	391	51.7%	202	26,7%	182	24,1%	1	0,1%
*Number of relapses (0 -5)*								
0	236	33.0%	0	0,0%	139	19,4%	1	0,1%
1	172	24.1%	1	0,1%	87	12,2%	0	0,0%
2	86	12.0%	2	0,3%	39	5,5%	0	0,0%
3	219	30.6%	3	0,4%	105	14,7%	2	0,3%
5	2	0.3%	5	0,7%	2	0,3%	0	0,0%
*Education*								
Primary School	23	2.0%	12	1,1%	6	0,5%	0	0,0%
Secondary School	239	20.9%	82	7,2%	80	7,0%	2	0,2%
High School	585	51.3%	206	18,1%	182	16,0%	1	0,1%
Bachelor	236	20.8%	60	5,3%	65	5,7%	0	0,0%
PhD, Master Degree	44	3.8%	16	1,4%	8	0,7%	0	0,0%
Other	13	1.1%	2	0,2%	2	0,2%	0	0,0%
*Employment*								
Full time job	510	48.8%	177	16,9%	164	15,7%	1	0,1%
Part time job	198	18.9%	46	4,4%	46	4,4%	0	0,0%
Retired	121	11.6%	42	4,0%	37	3,5%	1	0,1%
Unemployed	193	18.5%	73	7,0%	60	5,7%	1	0,1%
Other	23	2.3%	10	1,0%	13	1,2%	0	0,0%
*Children*								
No	327	44.7%	179	24,4%	145	19,8%	0	0,0%
Yes	406	55.3%	196	26,7%	198	27,0%	3	0,4%
Years from diagnosis	Total		Chron's disease		Ulcerative colitis		Unclassified colitis	
Mean	12.0		12.1		11.7		12.3	
Median	10		10		9.00		7	
Standard deviation	9.24		9.55		8.83		11.9	
Minimum	0		0		1		4	
Maximum	48		48		48		26	
*Age*								
Mean	53		57		59		61.0	
Median	43.0		42.5		45		59	
Standard deviation	24		31		25		20.1	
Minimum	18		17		16		42	
Maximum	93.8		82		83		82	

Concerning the patient health engagement level, measured through the PHE-s®, about the half of the sample (47%) reported a low health engagement level (Blackout or Alert) (see Table 2). Moreover, 30% of the sample globally reported a low level of HRQoL: particularly, 29% reported problems concerning systemic symptoms and social functioning, and 23% felt impaired because of bowel symptomatology.

**Table 2:** Distribution of PHE-s® levels (%) in the study sample

PHE-s® scores			
Low level of Patient Engagement		High level of Patient Engagement	
Blackout	Alert	Adhesion	Eudaimonic Project
7%	40%	42%	11%

Nevertheless, the main issue for patients of this study seems to be the psycho-emotional functioning, that is considered the aspect of HRQoL most impacted by IBD in 37% of the sample (see Table 3).

**Table 3:** Health-related Quality of Life levels (%) in the four domains assessed by the SIBD-Q in the study sample

	SIBD-Q scores	
	Low level of Health-related Quality of Life (< median value)	High level of Health-related Quality of Life (> median value)
Psycho-Emotional Functioning	37%	63%
Bowel Symptoms	23%	77%
Social Functioning	29%	71%
Systemic Symptoms	29%	71%

Moreover, regarding socio-demographic and clinical variables and their association with HRQoL, the number reported disease relapses showed a negative association with the SIBD-Q: the higher number of relapses are referred, the lower SIBD-Q is, concerning total score (r = -0.403; *p* < 0.001), systemic symptoms (r = -0.403; *p* < 0.001), bowel symptoms (r = -0.359; *p* < 0.001), social (r = -0.403; *p* < 0.001) and psycho-emotional (r = -0.334; *p* < 0.001) functioning. Gender was significantly associated to HRQoL: male patients reported higher mean values of HRQoL than female patients. This effect is significant for SIBD-Q total score (F = 14,67; *p* < . 01), as well as systemic symptoms (F = 13,35; *p* < . 01), emotional functioning (F = 9,26; *p* < . 05) and bowel-related symptoms (F = 14,93; *p* < . 01) but not for social functioning. To understand the relationship between the level of patient health engagement (PHE-s®) and the level of patients’ HRQoL (SIBD-Q) and if this relationship was affected by confounding variables—patients’ age, years from diagnosis, number of relapses, presence of children education level and employment—a multivariate analysis of covariance (MANCOVA) has been performed. Table 4 shows results of the MANCOVA. MANCOVA showed that PHE-s® has a statistically significant association with SIBD-Q total score and subscales when all confounding variables are controlled (F = 95,692; *p* < 0.01) as well as with all SIBD-Q subscales. Nevertheless, the interaction between PHE-s®levels and gender is not statistically significant (*p* > *0.05)*. Similarly, the interaction between PHE-s®and the number of relapses in the last 12 months does not show any significance (*p* > *0.05).*

**Table 4:** The MANCOVA model explaining PHE-s® and SIBD-Q association and the influence of socio-demographic and clinical variables. Only statistically significant socio-demographic and clinical variables have been included in the table

	Dependent Variable	Sum of Squares	df	Mean Square	F	p
PHE-s® Levels	SIBD-Q Total	264.66	3	88.221	95.629	< .001
	SIBD-Q Systemic symptoms	286.73	3	95.575	94.472	< .001
	SIBD-Q Social Functioning	293.71	3	97.905	56.848	< .001
	SIBD-Q Bowel Symptoms	233.65	3	77.882	55.074	< .001
	SIBD-Q Emotional functioning	308.10	3	102.700	84.294	< .001
Gender	SIBD-Q Total	13.54	1	13.536	14.672	< .001
	SIBD-Q Systemic symptoms	13.51	1	13.506	13.351	< .001
	SIBD-Q Social Functioning	4.14	1	4.138	2.402	0.122
	SIBD-Q Bowel Symptoms	21.12	1	21.123	14.937	< .001
	SIBD-Q Emotional functioning	11.29	1	11.292	9.269	0.002
Relapses in the last 12 moths	SIBD-Q Total	69.03	4	17.259	18.708	< .001
	SIBD-Q Systemic symptoms	79.19	4	19.796	19.568	< .001
	SIBD-Q Social Functioning	140.76	4	35.191	20.433	< .001
	SIBD-Q Bowel Symptoms	73.76	4	18.439	13.039	< .001
	SIBD-Q Emotional functioning	45.61	4	11.403	9.359	< .001
PHE-s®_Levels * Gender	SIBD-Q Total	3.39	3	1.130	1.225	0.300
	SIBD-Q Systemic symptoms	2.87	3	0.956	0.945	0.418
	SIBD-Q Social Functioning	1.40	3	0.467	0.271	0.846
	SIBD-Q Bowel Symptoms	2.56	3	0.854	0.604	0.613
	SIBD-Q Emotional functioning	5.85	3	1.951	1.601	0.188
PHE-s®_Levels * Relapses in the last 12 moths	SIBD-Q Total	9.09	10	0.909	0.986	0.455
	SIBD-Q Systemic symptoms	10.56	10	1.056	1.044	0.405
	SIBD-Q Social Functioning	20.15	10	2.015	1.170	0.309
	SIBD-Q Bowel Symptoms	14.52	10	1.452	1.027	0.419
	SIBD-Q Emotional functioning	8.13	10	0.813	0.667	0.755
Gender * Relapses in the last 12 moths	SIBD-Q Total	6.51	3	2.171	2.353	0.071
	SIBD-Q Systemic symptoms	6.84	3	2.281	2.255	0.081
	SIBD-Q Social Functioning	9.83	3	3.276	1.902	0.128
	SIBD-Q Bowel Symptoms	9.61	3	3.204	2.266	0.080
	SIBD-Q Emotional functioning	5.04	3	1.682	1.380	0.248
PHE-s®_Levels * Gender * Relapses in the last 12 moths	SIBD-Q Total	7.67	7	1.096	1.188	0.308
	SIBD-Q Systemic symptoms	7.29	7	1.041	1.029	0.410
	SIBD-Q Social Functioning	13.00	7	1.857	1.078	0.376
	SIBD-Q Bowel Symptoms	5.14	7	0.734	0.519	0.820
	SIBD-Q Emotional functioning	15.52	7	2.216	1.819	0.081
Residuals	SIBD-Q Total	466.80	506	0.923		
	SIBD-Q Systemic symptoms	511.91	506	1.012		
	SIBD-Q Social Functioning	871.45	506	1.722		
	SIBD-Q Bowel Symptoms	715.55	506	1.414		
	SIBD-Q Emotional functioning	616.49	506	1.218		

Finally, a linear regression model has been run to understand the relationship that occurs between patient health engagement scores (PHE-s®) and patients’ HRQoL scores (SIBD-Q). Consistently with our hypothesis, this analysis pointed out that PHE-s® is significantly related to the SIBD-Q total score (B = 0.585; *p* < 0.001; R squared = 0.343) and to the subscales’ scores—systemic symptoms (B = 0.572; *p* < 0.001; R squared = 0.327), bowel symptoms (B = 0.482; *p* < 0.001; R squared = 0.232), social (B = 0.485; *p* < 0.001; R squared = 0.234) and psycho-emotional (B = 0.607; *p* < 0.001; R squared = 0.369) functioning (see Table 5).

**Table 5:** Regression values of Patient Health Engagement (PHE-s®) on IBD patients' HRQoL (SIBD-Q)

	SIBD-Q									
	Psycho-Emotional Functioning		Systemic symptoms		Bowel Symptoms		Social Functioning		Total Score	
PHE-s®	B	R squared	B	R squared	B	R squared	B	R squared	B	R squared
	.607	.369	.572	.327	.482	.232	.485	.234	.585	.343

## Discussion

This manuscript is based on a cross-sectional study undertaken among patients with IBD and the participants are members of a voluntary-based patient organization, AMICI. The aim of this study was to assess the effects of IBD patient's level of health engagement on their HRQoL. The Italian version of the PHE-s®scale was used to evaluate patients' level of health engagement. The SIBD-Q was administered to assess the HRQoL. The association between HRQoL and patient health engagement was assessed by performing MANCOVA and linear regression models. The main finding of this study is that patient health engagement, considered as the patients’ psychological readiness to play an active role in the healthcare process [29], is significantly associated with the level of patients’ HRQoL when socio-demographic and clinical confounding variables are controlled. Since PHE-s® level has been found to be related with SIBD-Q, we deepened the nature of this relationship by running a regression analysis. The regression model confirmed, in line with our hypothesis, the significant association of patient engagement and patients’ HRQoL levels after controlling for the other confounding variables such as demographics and clinical ones. This result is in line with other studies—conducted on different clinical populations—that have demonstrated how playing an active role in the self-management of the disease can be useful to improve many aspects concerning both patients’ HRQoL and disease severity [52, 53]. For example, Druss and colleagues [54] tested an intervention aimed at sustaining patients with mental health issues in their involvement in the healthcare process. In that study, experimental group showed significantly greater improvements in patient engagement than those in usual care, along with greater improvements in adherence, physical activity, and HRQoL. Barnes and colleagues have discovered that higher patient engagement in their care is associated with remission in patients with IBD [55].

Moreover, according to existing literature about factors impacting on IBD patients’ HRQoLof life, this study confirmed the significant association of socio-demographics (i.e. gender, age) [47, 48] and clinical variables (i.e. reported disease relapses) [49] with IBD patients’ HRQoL. Our results, appears in line with the ones by Sainsbury and colleagues [17] whose research underlined that not only clinical factors, but also socio-demographics—including gender and age—appear to be important in affecting patients’ HRQoL, suggesting that clinicians’ attention needs to be drawn towards those variables to improve efficiently IBD patients’ HRQoL. Some authors, moreover, reported in their studies that IBD patients’ HRQoL was lower in patients who had hospitalization and more severe disease [50, 51]. Similarly, we found a correlation between the patients who reported many relapses and their HRQoL.. This supports that disease severity have an impact on HRQoL The discovery that patient health engagement has a significant relationship with patient’s HRQoL is one of the main strengths of this study and could have interesting practical implication in the development of interventions dedicated to enhancing patients’ wellbeing. Contrarily to socio-demographic and clinical characteristics that are factors more stable and difficult to modify, patient health engagement level can be modifiable through specific healthcare interventions. In this sense, as this is a modifiable factor, screening for patient engagement levels, coupled with appropriate interventions to improve this psychosocial aspect, could improve care, and ultimately improve HRQoL outcomes among IBD patients [56].

In this sense, the level of patient health engagement can be considered as a precious element to rely on, both for predicting IBD patients’ HRQoL and for modifying it, to sustain patients’ wellbeing.

However, our study also has limitations. Given that the A.M.I.C.I. onlus is a voluntary-based patient organization, participants of this study (which were selected among those individuals belonging to the patient organization) may show higher levels of patient health engagement than the general IBD population, which may limit the generalizability of our results. Moreover, it is possible that patients in AMICI could be more prone to be active in their own disease in the first place, considering they are part of a patient organization. Another limitation is that—due to privacy issues—we have no data about who did not fill out the questionnaire and thus we cannot compare the characteristics of this population with the one that constituted our study sample. Moreover, we had not the possibility to know the whole number of patients who were firstly contacted to fill out the survey. Furthermore, even if with a limited number, our sample included people diagnosed with unclassified colitis. Further research should consider collecting more precise data about this diagnosis (i.e. number of patients with proctitis, left-sided and pancolitis). Finally, although the population of the study was recruited in all the Italian regions, we did not have access to other geographic or socioeconomic factors that may also influence patient engagement and/or patients’ HRQoL such as urban or rural contexts or income.

## Conclusion

Patient health engagement is a novel concept in IBD research, and the study results suggest that IBD patients’ attitudes to play an active role in their healthcare may be a crucial factor in shaping HRQoL outcomes of this clinical population. Thus, our findings underline the importance of promoting patient health engagement initiatives to sustain patients’ HRQoL. Policy makers and clinicians—according to this study’s results—should increasingly recognize that patients can safeguard their HRQoL by being actively engaged in their health care. Indeed, successful patient health engagement is associated with a positive shift in the ways in which patients adjust to their illness condition and relate with their healthcare services, thus ultimately improving their own HRQoL.

## Data Availability

The datasets generated and/or analyzed during the current study are not publicly available due to data privacy reasons but are available from the corresponding author on reasonable request.

## Abbreviations

UC: Ulcerative Colitis
CD: Chron’s Disease
IBD: Inflammatory Bowel Disease
PHE-model: Patient Health Engagement Model
PHE-s®: Patient Health Engagement Scale
SIBD-Q: Short Inflammatory Bowel Disease Questionnaire
A.M.I.C.I. Onlus: Associazione Nazionale per le Malattie Infiammatorie Croniche dell’Intestino
